# Commentary on ‘Physics-informed deep learning parameterization of ocean vertical mixing improves climate simulations’ by Zhu *et al.*

**DOI:** 10.1093/nsr/nwac092

**Published:** 2022-05-18

**Authors:** Gustau Camps-Valls

**Affiliations:** Image Processing Laboratory (IPL), Universitat de València, Spain

Climate models constitute an essential tool to understand our planet, as they implement the laws of physics describing the ocean, land and atmosphere dynamics. However, resolving processes at fine resolutions constitute an important computational bottleneck. Parameterization (or closure) approximates such (e.g. turbulent) processes that cannot be resolved in climate models. Parameterizations rely on semi-empirical physical principles and typically improve modeling when included in coarse resolution climate models. However, they are not perfect and induce large biases in, e.g. ocean currents and heat. Machine learning algorithms could help alleviate the problem when massive amounts of observational data are available, but this is not typically the case.

**Figure 1:**
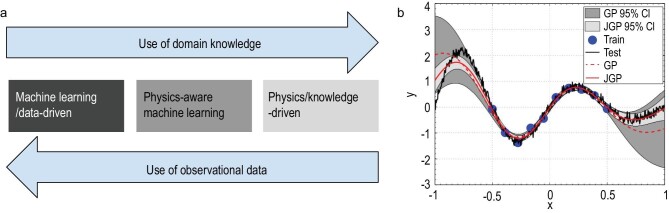
**Figure 1.** The concept of physics-aware machine learning. (a) When observational data is massively available for the whole representation domain, machine learning models suffice to approximate arbitrarily complex functions. Otherwise, purely physics-driven modeling exploits domain knowledge to approximate reality using semi-empirical mechanistic models. In the interplay, physics-aware machine learning exploits the regularities in the available data and constrains the model with physical constraints. (b) Physics-aware ML models generally improve physical consistency and generalization as they are able to operate in out-of-sample distributions. Unlike a standard Gaussian process (GP; red dashed line), a joint Gaussian process (JGP; red solid line) [2] that combines observational training data (blue circles) and model simulations (black solid line) allows us to predict well in the out-of-sample regime and provide more credible confidence intervals of the predictions that can be taken as extrapolation indicators.

Combining physics and machine learning promises advantages in generalization, consistency and extrapolation (see Fig. 1). The field was formalized in [1], illustrated for remote sensing [2,3] and turbulence ocean parameterization [4], and recently applied in climate attribution studies [5]. Answering to the urgent need to reduce uncertainties in vertical mixing parameterizations, Zhu *et al.* [6] deployed physics-informed mixing parameterization with a constrained neural network that learns knowledge directly from the turbulence observations while incorporating traditional physics-driven parameterization. The beauty of the method lies in its simplicity, which only requires appending simulations from the profile parameterization (PP) relation [7]:

(1) \begin{equation*} K_T = f(\rho ,N^2,U,S^2) + n \quad \rm {(standard\, ML\, approach)}, \end{equation*}

(2) \begin{eqnarray*} K_T \, = g(\rho, N^2,U,S^2,R_i^*) + n\\ \rm{such \, that}\quad K_T^* \,=\, h(R_i^*) \quad \rm{(physics-guided\, ML\, approach)}.\\ \end{eqnarray*}

Here ρ is the density, *N*^2^ the stratification, *S*^2^ the squared shear and *U* the velocity, while *f* and *g* are the neural networks learned, the relation *h* is a semi-empirical PP relation [7] used to generate additional data $\lbrace K_T^*,K_i^*\rbrace$ over the whole domain and $R_i^*=S^2/N^2$; cf. Fig. 1.

The model is not only able to provide excellent parameterizations but also shows a certain degree of extrapolation/generalization in predicting *K*_*T*_ below 150 m, where training data were non-existent. This was only possible when incorporating the physical constraint *h*. Besides, the authors in Zhu *et al.* [6] have observed enhanced ocean temperature simulations when the parameterization was employed in ocean-only and in complex atmosphere–ocean coupled climate modeling: improved modeling of turbulent heat flux provided more realistic simulations of the ocean thermal structure, while in the coupled climate model setting, improvements in temperature simulations of the tropical Pacific were also observed, with reductions in bias as high as 30% in the estimation of the Pacific cold tongue.

The field of deep learning parameterizations is becoming quite mature [4], and the work of Zhu *et al.* [6] contributes in a very practical way. Relying on simulations to enrich the training set is a convenient approach, typically referred to as ‘augmentation’ in the deep learning community. Alternative more sound approaches rooted in the concept of hybrid modeling [1] could have been applied: e.g. including the constraint *h* explicitly as a dependence regularizer [5], optimizing the relative relevance of observations and simulations [2] or even learning the parameters of *h* end to end [8]. Living in the physics-machine learning interplay is exciting these days but relevant challenges still need to be addressed: better generalization skills and extrapolation indicators are needed, model identifiability (equifinality) can be compromised when constraining the learning with (potentially weak or misspecified) physics models, and deep neural network interpretability and uncertainty quantification are urgent needs. Interdisciplinary approaches like this are one more step toward an integrative science where data-driven and knowledge-driven models coexist and cooperate.

***Conflict of interest statement.*** None declared.
